# Report: central diabetes insipidus and schwannoma in a male with X-linked congenital adrenal hypoplasia

**DOI:** 10.1186/s12902-020-00553-0

**Published:** 2020-05-27

**Authors:** Boo Kyeong Seo, Seul Ah. Jeong, Jae Young Cho, Ji Sook Park, Ji-Hyun Seo, Eun Sil Park, Jae-Young Lim, Hyang-Ok Woo, Hee-Shang Youn

**Affiliations:** 1 Department of Pediatrics, Gyeongsang National University School of Medicine, 92 Chilam-dong, Jinju, Gyeongnam 660-751 South Korea; 2 Gyeongsang Institute of Health Science, Jinju, Korea

**Keywords:** Adrenal hypoplasia congenita, Central diabetes insipidus, Schwannoma

## Abstract

**Background:**

*DAX1* mutations are related to the X-linked form of adrenal hypoplasia congenita (AHC) in infancy and to hypogonadotropic hypogonadism (HH) in puberty. We report a male patient affected by X-linked AHC who presented with central diabetes insipidus and schwannoma in adulthood, which has not been described in association with AHC.

**Case presentation:**

A 36-day-old male infant who presented with severe dehydration was admitted to the intensive care unit. His laboratory findings showed hyponatremia, hyperkalemia, hypoglycemia, and metabolic acidosis. After hormonal evaluation, he was diagnosed with adrenal insufficiency, and he recovered after treatment with hydrocortisone and a mineralocorticoid. He continued to take hydrocortisone and the mineralocorticoid after discharge. At the age of 17, he did not show any signs of puberty. On the basis of a GnRH test, a diagnosis of HH was made. At the age of 24, he was hospitalized with thirst, polydipsia and polyuria. He underwent a water deprivation test for polydipsia and was diagnosed with central diabetes insipidus. By quantitative polymerase chain reaction analysis, we identified a hemizygous frameshift mutation in *DAX1* (c.543delA).

**Conclusions:**

We suggest that *DAX1* mutations affect a wider variety of endocrine organs than previously known, including the posterior pituitary gland.

## Background

Adrenal hypoplasia congenita (AHC, OMIM 300200), which is caused by mutation of the dosage-sensitive sex reversal (DSS)-adrenal hypoplasia critical region on chromosome X, gene 1 (*DAX1*) gene, results in adrenal failure in males. Males with AHC often do not undergo puberty owing to hypogonadotropic hypogonadism (HH) caused by the same mutated *DAX1* gene [1].

The *DAX1* protein, also known as human nuclear receptor subfamily 0, group B, member 1 (*NR0B1*), is encoded by an X-linked gene that acts at multiple levels in the development of the adrenal glands, hypothalamus, pituitary, ovaries, and testes and is expressed in these tissues during development and postnatal life. However, the exact biological role of *DAX1* is unknown [1–5]. HH is thought to be likely due to developmental defects in the hypothalamus and pituitary gland, suggesting a role for *DAX1* in the development of these organs.

The objective of this case report is to show, likely for the first time, that *DAX1* is a survival signal or is related to maintenance of the posterior pituitary gland. To our knowledge, no such findings have been reported previously in the literature. Our evidence also suggests that in humans, *DAX1* plays multiple roles in the development and maintenance of several endocrine organs.

## Case presentation

A 36-day-old male infant was admitted to the intensive care unit (ICU) with severe lethargy, tachypnea, severe dehydration, 12% weight loss since birth, diarrhea, and fever (38 °C). His prenatal and birth history (term; birth weight, 3500 g) were unremarkable, and he exhibited normal male genital development. The biochemical measurements showed hyponatremia (Na, 126 mmol/L), normochloremia (Cl, 100 mmol/L), hyperkalemia (K, 10.8 mmol/L), and hypoglycemia (glucose, 50 mg/dL). He also had metabolic acidosis due to diarrhea. Arterial blood gas analysis showed a pH of 7.17, a carbon dioxide partial pressure of 24 mmHg (reference range: 35–45), and a bicarbonate concentration of 8.9 mEq/L (reference range: 22–26). The low plasma bicarbonate concentration of 8.9 mEq/L (15.1 mEq/L lower than normal levels) was associated with the reduced carbon dioxide partial pressure of approximately 24 mmHg. The patient was given 20 cc/kg of fluid with 5% dextrose, sodium chloride, and sodium bicarbonate for 1 h followed by maintenance fluid. Antibiotics (cefotaxime and gentamicin) were administered after a diagnosis of sepsis. After the initial interventions, his general condition seemed to be recovered, although hyponatremia and hyperkalemia persisted (Na, 128 mmol/L; K, 6.7 mmol/L). Further biochemical investigation showed an extremely high adrenocorticotropic hormone (ACTH) level (2000 pg/mL; reference range: 0–10.12 pmol/mL), high plasma renin activity (16.8 μg/mL/hr.; reference range: 0.32–1.84 μg/mL/hr) and a low aldosterone level (0.69 ng/dL; reference range: 2.0–110.0 ng/dL). He seemed to exhibit clinical decompensation after being in a highly fragile condition. A stressor (in the case, the infection) seemed to trigger an adrenal crisis.

The patient’s karyotype was 46,XY. His 17-hydroxyprogesterone level (0.83 ng/mL; reference range: 0.7–2.5 ng/mL) and testosterone level (0.95 ng/mL; reference range: < 1.77 ng/ml) were normal, so we excluded congenital adrenal hyperplasia. The child was diagnosed with adrenal insufficiency and administered 6 mg of hydrocortisone and 0.1 mg of fludrocortisone (Florinef) once daily. His electrolyte imbalance and hypoglycemia were also corrected (arterial blood gas analysis: pH 7.34, PCO_2_ 37 mmHg, HCO_3_ 20 mmol/L; Na, 137 mmol/L; K, 5.7 mmol/L; glucose, 90 mg/dL).

The patient was required to maintain glucocorticoid and mineralocorticoid treatment after discharge. We informed the patient’s parents that the glucocorticoid should be administered at an increased dose during stressful situations such as surgery, inflammation and trauma. Because his parents’ medication compliance was low, the patient was frequently hospitalized for adrenal crisis.

**Fig. 1:**
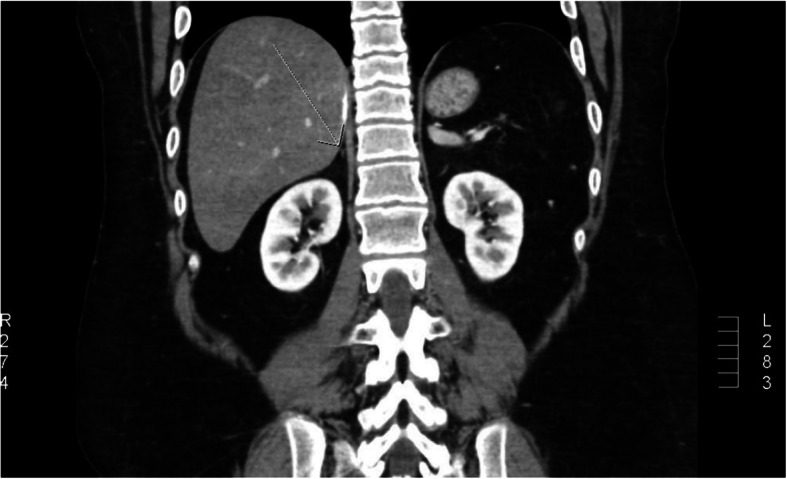
Abdominal CT scan results showing severely atrophied adrenal glands (arrow)

At the age of 17 years, the patient showed no signs of puberty and had no axillary or pubic hair (Tanner stage 1). His basal gonadotropin levels were measured; the LH level was 0.40 mIU/mL, and the FSH level was 3.26 mIU/mL. A GnRH test (Relefact, 0.1 mg, Aventis Pharma, gonadorelin acetate) was performed, which showed prepubertal gonadotropin peak levels (LH 1.07 mU/mL, FSH, 3.58 mU/mL). These results, together with the lack of any sign of puberty, were consistent with the diagnosis of HH. The patient was given testosterone replacement therapy, which induced clinical signs of puberty, including a growth spurt. Other hormone levels were also tested. The results of the thyroid function test were in the normal ranges (T3 11.40 ng/dL, TSH 0.72 mIU/L, free T4 1.27 ng/dL). The growth hormone axis test results were also within normal ranges (insulin-like growth factor 1159.0 ng/mL;reference range: 57–426 ng/mL, basal growth hormone 0.26 ng/mL; reference range: 0.18–9.76 ng/mL). Abdominal computed tomography conducted at the time HH was diagnosed revealed severe atrophy of both adrenal glands (Fig. 1).

**Fig. 2:**
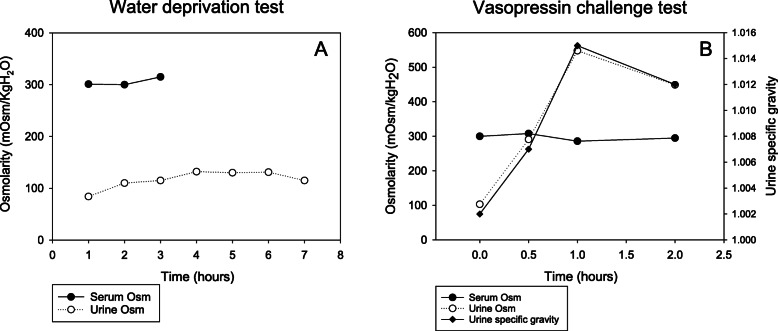
Water deprivation test results (**a**). Vasopressin challenge test results (**b**). There was no change in urine osmolality during the water deprivation test, while a greater than fivefold increase in urine osmolality was observed after administration of vasopressin

**Fig. 3:**
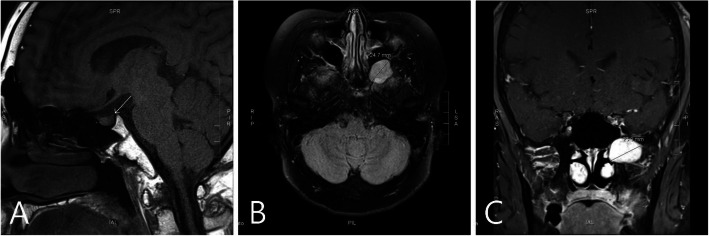
Magnetic resonance image of the sella turcica. Sagittal T1W1 (**a**). Axial T2W1 (**b**). Coronary enhanced T1W1 (**c**). Loss of normal T1 hyperintensity was observed in the posterior lobe of the pituitary gland (arrow). An enhanced 2.5-cm mass suspected of being a schwannoma is visible in the area of the left pterygopalatine fossa

At the age of 24, the patient’s height was 180 cm (in approximately the 75th centile). He continued to have very low levels of arginine vasopressin (AVP, 1.47 pg/mL) and exhibited hypernatremia (146.1 mmol/L) and persistent strong thirst. He presented with polydipsia and polyuria (7 L/day), and his urinalysis showed low specific gravity (1.002) and low urine osmolarity (54 mOsm/kg H_2_O). Because his serum glucose and HbA1c levels were in the normal range, we ruled out diabetes mellitus. He underwent a water deprivation test, and the results revealed that the urine was not concentrated based on osmolality and that the urine output and serum sodium level were not changed, thus excluding primary polydipsia (Fig. 2a). We then conducted a vasopressin challenge test to check for central diabetes insipidus and found that the patient’s urine was five times more concentrated than normal according to osmolality. Subsequently, the patient was diagnosed with central diabetes insipidus (Fig. 2b). Magnetic resonance imaging showed a loss of signal in the posterior pituitary gland and an abnormal mass in the maxillary sinus (Fig. 3). The signal changes in the posterior pituitary gland were consistent with central diabetes insipidus. After diagnosis, the symptoms were controlled with desmopressin spray (15 μg per dose twice a day). Because there has been no change in size, the left maxillary sinus mass is presumed to be a schwannoma and is being observed every 6 months without excision. We shared the diagnosis and treatment plan with the patient.

**Fig. 4:**
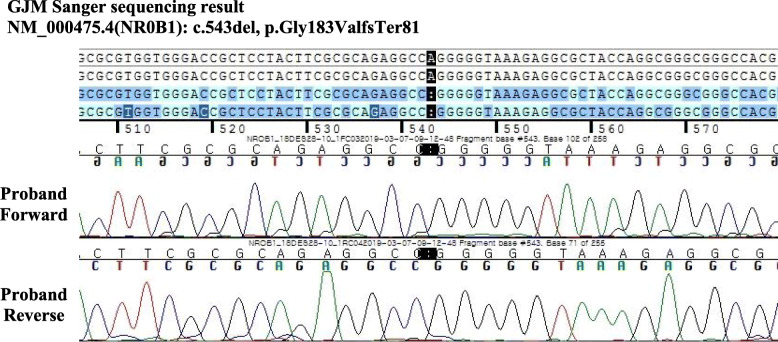
Identification of a hemizygous missense mutation in the *DAX1* gene located at Xq21. The sequencing chromatogram shows a point mutation of c.543del. This mutation leads to the replacement of glycine with valine at the 183rd residue, triggering early termination

An outside laboratory then conducted quantitative polymerase chain reaction (PCR) analysis to identify mutations in *DAX1*. PCR revealed that a base located at position 543 on gene *DAX1*, the causative gene of AHC, was deleted, which caused the 183rd amino acid, glycine, to be replaced with valine. This mutation was a frameshift mutation resulting in replacement of the 81st amino acid codon with a stop codon, which induced a loss of function (Fig. 4). The initial sequencing results (at 17 years old) were misread, but our hospital’s molecular diagnostic team found the errors when they reread the PCR sequencing results.

In summary, this male exhibited no genital abnormality at birth and was determined to be 46,XY with adrenal insufficiency, HH and central diabetes insipidus, consistent with the diagnosis of AHC, despite the unusual presence of diabetes insipidus. For maintenance, he was prescribed hydrocortisone, a mineralocorticoid and vasopressin.

## Discussion and conclusions

This is the first report of central diabetes insipidus in adulthood in an AHC patient, suggesting that the *DAX1* gene in AHC affects more endocrine organs than previously believed.

Three patients with a hemizygous frame shift mutation in *DAX1* (c.543delA) have been described in the literature pertaining to AHC. In addition, we are aware of another AHC patient with the same mutation as our patient [6]. That patient was delivered at 38 weeks of gestational age with a birth weight of 3160 g. He was admitted to the neonatal ICU at birth because of neonatal respiratory distress syndrome. After exogenous pulmonary surfactant replacement, he recovered and was discharged. However, 7 days after discharge (on postnatal day 18), he returned to the hospital because of poor weight gain and severe dehydration. Laboratory results revealed primary adrenal insufficiency, and he was diagnosed with AHC due to the same mutation (c.543delA) of the *DAX1* gene that was found in our patient. That child is now 12 years old. His younger brother has the same mutation in *DAX1* but has not yet shown symptoms of central diabetes insipidus.

*DAX1* is expressed in tissues and cells involved in steroid hormone production and reproductive function, including the adrenal cortex, testicular Leydig and Sertoli cells, ovarian theca and granulosa cells, pituitary gonadotropes, the ventromedial hypothalamic nucleus in the brain and some other brain areas (arcuate nuclei, amygdala, hippocampus, cerebral cortex) [3, 4, 7, 8].

AVP is synthesized in the hypothalamus. Specifically, it is principally produced by neurons whose cell bodies are within the supraoptic nuclei of the hypothalamus. It is also produced, albeit in smaller quantities, in neurons with cell bodies located in the paraventricular nuclei, the primary sites of production of oxytocin, a homologous hormone primarily involved in uterine contraction and milk letdown. Storage vesicles are transported down neuronal axons through the hypothalamic-hypophysial tract and are ultimately released in the posterior pituitary gland [9, 10].

However, the mechanisms of *DAX1* action in the hypothalamus and pituitary gland during development and adult function remain unknown. More studies are needed to determine the exact roles of *DAX1* in the hypothalamus and pituitary gland.

Two cases of *DAX1* mutations have been reported in association with hypothalamic and pituitary defects in gonadotropin production [11].

*DAX1* may play pleiotropic roles during the development and adult functioning of the hypothalamic-pituitary-adrenal-gonadal axis. If so, *DAX1* mutations could cause various complex endocrine phenotype defects [2, 11].

There have also been reports that *DAX1* expression is related to cancer. As we have previously observed in an animal study, *DAX1* is expressed in various brain tissues [12–15]. Notably, our patient’s younger sister was diagnosed with pontine glioma at the age of 5 years. We believe that her brother’s schwannoma may be related to *DAX1* mutation *and* to cancer originating from brain tissues. In AHC patients, *DAX1* is mutated at the germline level. Thus, more studies are needed to determine cancer prevalence and type among these patients.

It is also possible that *DAX1* functions as a repressor that controls the rate of stem cell differentiation during organ development [16]. Premature differentiation of pluripotent stem cells into mature cells without prior expansion of cell numbers could lead to transient overactivity and then to subsequent organ hypoplasia due to depletion of the pluripotent cell pool. A clinical case in which the axis was active from the infantile period to the age of 3 years supports this hypothesis [17]. Additional evidence for this hypothesis comes from a mouse model of *DAX1* exon 2 deletion [18]. This mouse model has been used to show that during the aging process, *DAX1*-deficient mice experience adrenal failure. However, it is possible that the numbers of stem cells could differ among species and endocrine tissues.

In conclusion, as the life expectancy of AHC patients increases with the development of improved treatment strategies, we expect more cases to be identified. We think our patient is the first example of such a case.

## Data Availability

Not applicable.

## Abbreviations

AHC: Adrenal hypoplasia congenita
HH: Hypogonadotropic hypogonadism
NR0B1: Nuclear receptor subfamily 0, group B, member 1
DAX1: Dosage-sensitive sex reversal-adrenal hypoplasia critical region on chromosome X, gene 1
SF1: Steroidogenic factor 1
